# Determination of Haematological Reference Ranges in Healthy Adults in Three Regions in Ghana

**DOI:** 10.1155/2019/7467512

**Published:** 2019-02-05

**Authors:** Otchere Addai-Mensah, Daniel Gyamfi, Richard Vikpebah Duneeh, Kwabena O. Danquah, Max E. Annani-Akollor, Lillian Boateng, Eddie-Williams Owiredu, Francis A. Amponsah, Edward Y. Afriyie, Renate Asare, David Ntiamoah Ofosu

**Affiliations:** ^1^Faculty of Allied Health Sciences, Kwame Nkrumah University of Science and Technology, Kumasi, Ghana; ^2^Department of Medical Laboratory Technology, Faculty of Allied Health Sciences, Kwame Nkrumah University of Science and Technology, Kumasi, Ghana; ^3^Department of Haematology, Komfo Anokye Teaching Hospital, Kumasi, Ghana; ^4^Department of Molecular Medicine, School of Medical Sciences, Kwame Nkrumah University of Science and Technology, Kumasi, Ghana; ^5^Saint John of God Hospital, Duayaw Nkwanta, Sunyani, Ghana

## Abstract

Laboratory results interpretation for diagnostic accuracy and clinical decision-making in this period of evidence-based medicine requires cut-off values or reference ranges that are reflective of the geographical area where the individual resides. Several studies have shown significant differences between and within populations, emphasizing the need for population-specific reference ranges. This cross-sectional experimental study sought to establish the haematological reference values in apparently healthy individuals in three regions in Ghana. Study sites included Nkenkaasu, Winneba, and Nadowli in the Ashanti, Central, and Upper West regions of Ghana, respectively. A total of 488 healthy participants were recruited using the Clinical and Laboratory Standards Institute (United States National Consensus Committee on Laboratory Standards, NCCLS) Guidance Document C28A2. Medians for haematological parameters were calculated and reference values determined at 2.5^th^ and 97.5^th^ percentiles and compared with Caucasian values adopted by our laboratory as reference ranges and values from other African and Western countries. RBC count, haemoglobin, and haematocrit (HCT) were significantly higher in males compared to females. There were significant intraregional and interregional as well as international variations of haematological reference ranges in the populations studied. We conclude that, for each geographical area, there is a need to establish geography-specific reference ranges if accurate diagnosis and concise clinical decisions are to be made.

## 1. Introduction

A reference range is a range of values of a laboratory test usually based on predetermined test results from a group of apparently healthy individuals and is critical for diagnostic accuracy [1]. Reference ranges in haematology are thus useful in results interpretation and in clinical decision-making in this era of evidence-based medicine. They are used as a guide in clinical trials to set inclusion and exclusion criteria and serve as the basis for safety monitoring for trial participants [2, 3]. Population-based haematological reference ranges have not been established for many healthcare facilities in Ghana unlike many developing countries [4]. Many of the reference ranges in use are those established in the countries of origin of the haematological analyzers in use. The danger accompanying this is the use of established reference ranges in western settings for populations that are diverse in social status, health, and geographical setting [5]. There are inter- and intrapopulation variation in haematological reference ranges even among populations of the same race and especially so in populations of varying genetics, pathogen sets, nutritional status, and altitude [6]. The variation makes dependence on preestablished haematological reference values from other countries inappropriate, thereby leading to misdiagnoses resulting in wrong treatment and its attendant dire health implications on individuals, families, communities, and the nation at large.

It is therefore expected that different parts (regions) of the country will have different reference ranges based on their peculiarities. Out of this, a set of national reference ranges may be established.

We hypothesized that haematological reference ranges have intra- and inter-population variation, differing from one population set to another and from one country to another.

This study, therefore, sought to establish the haematological reference values in apparently healthy voluntary nonremunerated blood donors from three regions in Ghana.

## 2. Methodology

### 2.1. Study Design, Site, and Population

This was a cross-sectional experimental study conducted between February and July 2018. Ghana has three major ecological zones: Savanna, Rain Forest, and Coastal zones. Participants were recruited from all three zones to establish region-specific reference ranges and to assess the interregional variabilities in haematological reference ranges in Ghana. Sites used for sampling are Nkenkaasu (Rain Forest), Winneba (Coastal), and Nadowli (Savanna) in the Ashanti, Central, and Upper West regions, respectively. Nkenkaasu is a town that lies along latitude 7.32336 and longitude -1.90260. It is located in the Ashanti Region in the Offinso Municipality with a population of 138,190. Winneba, the capital of Effutu Municipal district, on the other hand is located in the Central region of Ghana and lies on latitude 5.36223 and longitude -0.62989 with a total population of 55,331. It is located 56 km west of Accra and 140 km east of Cape Coast. Nadowli is located in the Upper West region of Ghana and lies on latitude 10.34104 and longitude -2.38136. It is one of the nine districts in the Upper West region and covers an area of about 2,594 km^2^ with a total population of 61,561 [7]. A total sample size of 600 participants (300 males and 300 females) was targeted for this study according to the Clinical and Laboratory Standards Institute Guidance Document C28A2 [8]. However, 488 participants consisting of 218 males and 270 females were recruited after excluding participants with confounding factors (G6PD deficiency, sickle cell disease, and asymptomatic malaria). All the study participants were from urban setting. Participant recruitment and sampling were done in the same season.

### 2.2. Ethical Considerations

Ethical approval for the study was obtained from the Committee on Human Research Publication and Ethics (CHRPE) of the School of Medical Sciences, Kwame Nkrumah University of Science and Technology (CHRPE/AP/483/17), and also from the Research and Development Department of Komfo Anokye Teaching Hospital (KATH). Written informed consent was obtained from all participants after the aims and objectives of the study had been explained to them.

### 2.3. Inclusion and Exclusion Criteria

Apparently healthy voluntary nonremunerated blood donors between the ages of 18 and 60 years who consented were included in this study. All females were not pregnant. Individuals with confounders like malaria, glucose-6-phospate dehydrogenase deficiency, and sickle cell disease were excluded.

### 2.4. Laboratory Analysis

5 ml of venous blood was collected from the antecubital fossa and dispensed into K3EDTA tubes (Becton Dickinson, Plymouth, UK). Screening for confounding factors for each participant was performed. 10% Giemsa-stained thick and thin films were prepared on clean grease-free slides (thin films fixed with absolute methanol) for malaria microscopy. G6PD screening was performed with methaemoglobin reductase technique as described by Brewer et al. [9]. 1 ml of well-mixed anticoagulated whole blood was put into three test tubes labelled* test*,* positive*, and* negative*, respectively. 50 *μ*l of 1.25% NaNO3/5% glucose mixture was then pipetted into the tubes labelled* test* and* positive* and 50 *μ*l of 0.015% methylene blue was pipetted into the tubes labelled* test* and* negative*. The mixture was well mixed, corked, and incubated in the water bath at 37°C for 3 hrs with hourly mixing. After the 3 hrs, 0.1 ml the content of each tube was pipetted into separately labelled tubes containing 10 ml of distilled water and the reading was done against a white background. Sickle cell screening was performed using the 2% sodium metabisulphite technique [10]. Whole blood was mixed with 2% sodium metabisulphite and incubated at room temperature for an hour. The slides were then mounted under light microscope and focused using low-power objective lens (10X), followed by examination under high-power lens (40X). Deoxygenation of the erythrocytic haemoglobin due to the reducing agent results in sickling of RBCs with the haemoglobin S variant. Haemoglobin phenotyping was also performed for each participant. Phenotyping was performed using alkaline electrophoresis. Tris EDTA with a pH of 8.6 [11] was used as buffer and analysis was done at 250V after 40 minutes.

The tests were performed immediately after sample collection to minimize storage variabilities. Blood samples of subjects who satisfied the inclusion criteria were then transported in cold blood sample tube transportation box (DM Innovators, Ahmedabad, India) to the Haematology Department of the Komfo Anokye Teaching Hospital (KATH) for further processing. At KATH, the samples were brought to room temperature, after which complete blood counts (CBC) with 5-part differential were performed using Sysmex KX 4000i haematology analyzer (Sysmex Corporation, Kobe, Japan). Sysmex KX 4000i uses the optical detector block to analyze WBCs and reticulocytes based on fluorescence flow cytometry using a semiconductor laser. RBCs and platelet count are analyzed using the Hydro-Dynamic Focusing and haemoglobin is analyzed based on the cyanide-free sodium lauryl sulphate (SLS) haemoglobin determination method [12]. CBC was performed within 8 hours of blood draw. Daily calibration and maintenance of the analyzer were performed according to the manufacturer's instructions. Internal quality control (QC) was performed and analyses began only when all quality controls passed. Precision of analysis was assessed by internal quality control and accuracy was determined based on external quality control performance. Uniformity of calibration was also ensured as all CBCs were performed using the Sysmex KX 4000i haematology analyzer at KATH. All laboratory tests were carried out by licensed Medical Laboratory Scientists.

### 2.5. Statistical Methods

Demographic data were collected using an investigator-administered questionnaire in a language that they could easily comprehend. Data was entered into excel and cleaned prior to analysis. All categorical data were presented as frequencies and percentages and reference ranges were calculated using nonparametric methods. Reference values were determined at 2.5^th^ and 97.5^th^ percentiles. The Mann-Whitney* U* test was used to determine the significance of differences between males and females. The Kruskal-Wallis test was used to determine the significance of differences between the three regions, Ashanti, Central, and Upper West region, followed by Bonferroni post hoc test for pairwise analysis.* P* value < 0.05 was considered significant. All statistical analyses were performed with IBM SPSS version 25.0.

## 3. Results

Table 1 shows the sociodemographic characteristics of the entire study population. A total of 488 apparently healthy voluntary nonremunerated blood donors with mean age of 32.08 ± 11.67 years and ages ranging between 18 and 60 years were recruited for the study. There were more females (55.3%) than males (44.7%). The highest number of participants was recorded from Upper West region, and majority of the participants had haemoglobin A (85.5%) (Table 1).

Haematological reference ranges of the study population stratified by gender are shown in Table 2. Almost all the haematological parameters estimated were lower than those stated in the accompanying manual of the heamatology analyzer used in the study. Males had statistically significant higher RBC count, haemoglobin concentration, HCT, MCH, MCHC, Lymphocyte Abs, Lymphocyte %, Monocyte %, Eosinophil %, and Basophil %, whiles females had elevated RDW-CV, Platelet, Neutrophil Abs, and Neutrophil % (Table 2).

Table 3 shows the haematological reference ranges of the study population stratified by regions. Participants from Ashanti region recorded statistically significant higher TWBC count, Basophil %, Neutrophil Abs, and Lymphocyte Abs and lower Monocyte % compared to both Central and Upper West regions but had lower Basophil Abs compared to Central region. Central region recorded a lower haemoglobin concentration, HCT, MCV, and MCH compared to the Ashanti and Upper West regions. The Upper West region had the highest haemoglobin level, HCT, MCV, and MCH. Central region also had the highest RDW-CV compared to Upper West region (Table 3).

## 4. Discussion

Most reference ranges that many African countries rely on are those established among Caucasian populations. Diversity in social and health status and geographical setting makes the dependence on these preestablished haematological reference ranges from other countries inappropriate, thereby leading to misdiagnoses resulting in wrong treatment.

This study, therefore, established the haematological reference values in apparently healthy populations from three regions in Ghana.

There were significant gender-related differences in the haematological reference ranges established in this study (Table 2). A similar observation was made when compared with the reference ranges in different countries [1, 13–16]. Reference ranges for RBC count, haemoglobin, and HCT were higher among males compared to females (Table 2). This finding is consistent with reports from other studies in Africa [1, 3, 5, 13, 14, 16, 17] as well as USA [18]. The platelet count was higher in women than in men, consistent with studies by Kibaya et al. in 2008 [14], Mine et al. in 2011 [16], and Miri-Dashe et al. in 2011 [1]. The gender-wise differences in these reference ranges may be attributed to the variations in the types of hormones produced and their corresponding concentrations in the different sexes as well as the effect of erythropoietin release in response to regular menstruation and cross-stimulating megakaryopoiesis [1, 3].

It was also observed in our study that the haematological reference ranges (Tables 2 and 3) were lower compared to the accompanying manual of the haematology analyzer used in the study. Reference ranges for RBC count, haemoglobin, and red cell indices (HCT, MCV, MCH, and MCHC) as well as platelet count were below the lower limit of the accompanying predetermined reference ranges. Similar observations have been made in Botswana [16], Kenya [14], Uganda [13], and other African countries [5, 19]. This implies that a percentage of these participants could be wrongly classified as having erythrocytopaenia, anaemia, and thrombocytopaenia using the reference values that are often quoted. The lower reference ranges for red cell indices in this study compared to those of Caucasians may be attributed to the relatively lower ferritin and transferrin saturation among blacks [20] as well as poor nutritional status among the general Ghanaian population [21]. Furthermore, that for platelet count could be attributed to genetic factors as well as increased consumption of platelets as a result of the high prevalence of malaria infection in Ghana [22, 23].

The reference ranges for total white blood cells including some differentials counts (Monocyte and Eosinophils) in this study were above the upper limit of the accompanying reference range. This may be due to the higher prevalence of parasitic infections and its associated leukocytosis and eosinophilia among Ghanaians [23, 24]

Moreover, significant interregional variation of haematological reference ranges was observed in this study (Table 3). The reference ranges with respect to TWBC, Neutrophil Abs, Neutrophil %, and Lymphocyte were higher among participants from Ashanti region. This may be due to the rising incidence of infections such as malaria in this region [25]. Additionally, the reference ranges for haemoglobin level, HCT, MCV, MCH, Lymphocyte %, Eosinophil Abs, Eosinophil, and Basophil were higher among participants from the Upper West region (Table 3). The increased red cell indices among the Upper West region is attributable to the region's dependence on natural organic products and, conversely, the increasing rate of processed food consumption among its southern counterparts [26]. However, the increased Eosinophil and Basophils could be linked to higher incidence of allergic conditions in the Northern part of Ghana [27]. Such interregional variation of haematological reference ranges has also been observed in studies by Koram et al. in 2007 among participants from the Akuapem North district in Ghana [4] and by Dosoo et al. in 2012 among subjects from the middle belt of Ghana [3]. Other factors that could contribute to these differences are environmental and genetic factors or a combination of both or several other factors such as lifestyle differences between these participants from these regions.

This study is limited by the fact that participants from only three major regions in Ghana were included and may not represent the haematological reference ranges of all of Ghana. Another limitation of this study is the fact that we did not assess the menstruation/menopausal status of females and were unable to physically assess volunteers for asymptomatic splenomegaly and other possible confounding conditions.

## 5. Conclusion

The laboratory reference ranges established in this study are one of the most comprehensive haematology data sets generated in Ghana. It is certain that lifestyle, physical, and genetic factors all affect the normal physiological processes of a population, and hence it is expected that there would be variations in the measurement of “normal” functions among and between populations. There is therefore a need to establish reference ranges that are specific to a particular population instead of applying reference values determined for one population on another population.

## Figures and Tables

**Table 1:** Sociodemographic characteristics of study population.

Variable	Mean ± SD	Range
Age (years)	32.08 ± 11.67	18-60
	*Frequency (n=488)*	*Percentage (*%)
*Sex*		
Female	270	55.3
Male	218	44.7
*Region*		
Ashanti	145	29.7
Central	168	34.4
Upper West	175	35.9
*Hb Phenotype*		
A	417	85.5
AC	59	12.1
AD	3	0.6
AF	4	0.8
C	4	0.8
D	1	0.2

**Table 2:** Haematological reference ranges of the study population stratified by gender.

Parameters	Unit	Males			Female			p-value
		N	Median	Reference values	N	Median	Reference values	
TWBC	10^3^/*μ*L	216	5.47	3.28-11.23	266	5.62	3.25-10.64	0.305
RBC	10^6^/*μ*L	216	5.19	3.61-6.97	266	4.38	3.08-5.88	**<0.0001**
Haemoglobin	g/dL	216	15.20	10.69-18.76	266	12.50	8.19-16.17	**<0.0001**
HCT	%	216	45.20	31.8-61.83	265	37.40	26.76-50.44	**<0.0001**
MCV	fL	216	87.05	69.71-103.23	266	86.80	64.44-103.53	0.226
MCH	pg	216	29.40	23.30-34.16	266	28.70	19.54-33.73	**0.002**
MCHC	g/dL	216	33.70	29.74-37.16	266	33.10	26.81-37.13	**<0.0001**
RDW-CV	%	216	14.00	11.70-18.66	263	14.30	11.80-26.40	**0.044**
Platelet	10^3^/*μ*L	216	186.00	85.93-348.20	265	214.00	110.95-416.30	**<0.0001**
Neutrophils Abs	10^3^/*μ*L	202	2.08	0.65-5.50	251	2.23	0.57-6.08	**0.031**
Neutrophils	%	202	39.55	17.57-67.46	252	44.75	20.93-74.47	**<0.0001**
Lymphocytes Abs	10^3^/*μ*L	211	2.35	0.77-4.78	264	2.25	0.64-4.28	**0.026**
Lymphocytes	%	211	45.70	11.98-66.91	264	41.25	14.59-62.25	**<0.0001**
Monocytes Abs	10^3^/*μ*L	211	0.51	0.21-1.02	264	0.48	0.19-1.02	0.286
Monocytes	%	211	9.69	4.30-15.17	264	8.80	4.55-17.18	**0.043**
Eosinophils Abs	10^3^/*μ*L	202	0.16	0.01-0.896	251	0.12	0.02-0.90	0.216
Eosinophils	%	203	3.00	0.21-13.91	251	2.40	0.33-14.30	**0.005**
Basophils Abs	10^3^/*μ*L	212	0.03	0.01-0.094	264	0.03	0.01-0.11	**0.010**
Basophils	%	212	0.60	0.10-1.94	264	0.50	0.10-2.14	**0.011**

Mann-Whitney *U* test was performed to compare males and females. P<0.05 was considered statistically significant (p-values of significant variable in bold print). TWBC: Total White Blood Cells; RBC: Red Blood Cells; HCT: Haematocrit; MCV: Mean Cell Volume, MCH: Mean Cell Haemoglobin; MCHC: Mean Cell Haemoglobin Concentration; RDW-CV: Red cell Distribution Width-Coefficient of Variation; Abs: Absolute.

**Table 3:** Haematological reference ranges of the study population stratified by regions.

Parameters	Ashanti Region (A)			Central Region (C)			Upper West Region (U)			p-value	Significant pairs
	N	Median	Reference values	N	Median	Reference values	N	Median	Reference values		
TWBC	145	6.03	3.56-12.64	168	5.32	3.12-10.47	171	5.34	3.35-9.86	**<0.0001**	A&C; A&U
RBC	145	4.61	3.34-6.07	168	4.53	2.99-7.7.01	171	4.76	3.54-6.67	0.083	-
Haemoglobin	145	13.10	9.09-16.51	168	12.70	7.49-20.71	171	14.30	9.57-18.33	**<0.0001**	A&C; C&U
HCT	145	39.40	26.83-52.41	168	38.60	25.72-63.12	170	42.76	30.51-57.00	**<0.0001**	A&C; C&U
MCV	145	86.30	66.25-102.75	168	84.60	61.73-104.31	171	90.00	70.03-102.61	**<0.0001**	A&C; C&U
MCH	145	28.60	21.55-33.20	168	28.40	19.18-34.28	171	30.00	22.36-34.71	**<0.0001**	A&C; C&U
MCHC	145	33.20	29.77-37.00	168	33.70	25.31-37.78	171	33.40	29.89-36.94	0.807	-
RDW-CV	144	14.00	11.96-19.89	166	14.45	11.82-33.03	171	13.90	11.70-17.77	**0.001**	C&U
Platelet	145	212.00	75.95-428.55	168	202.50	100.23-451.08	170	196.00	119.55-349.70	0.222	-
Neutrophils Abs	141	2.51	1.19-9.03	153	2.06	0.31-5.71	160	1.94	0.75-4.62	**<0.0001**	A&C; A&U
Neutrophils %	141	44.80	24.73-77.98	154	44.04	12.75-74.75	160	39.30	21.92-64.28	**<0.0001**	A&C; C&U
Lymphocytes Abs	141	2.53	0.87-5.80	167	1.93	0.52-4.02	160	2.34	0.93-4.02	**<0.0001**	A&C; A&U; C&U
Lymphocytes %	141	43.00	10.86-64.42	167	40.90	11.98-67.78	169	45.10	14.97-64.03	**0.020**	C&U
Monocytes Abs	141	0.51	0.24-1.30	167	0.46	0.12-0.99	169	0.51	0.19-0.89	**0.018**	A&U
Monocytes %	141	8.30	4.20-15.98	167	9.70	5.04-19.36	169	9.50	4.70-15.20	**0.005**	A&C; A&U
Eosinophils Abs	141	0.12	0.01-0.66	153	0.10	0.01-1.04	160	0.17	0.03-0.94	**0.025**	C&U
Eosinophils %	141	2.00	0.16-9.50	153	2.60	0.29-15.84	161	3.40	0.61-15.53	**<0.0001**	A&C; C&U
Basophils Abs	141	0.02	0.01-0.12	167	0.03	0.01-0.11	170	0.03	0.01-0.08	**0.009**	A&C
Basophils %	141	0.40	0.1-1.34	167	0.50	0.1-2.78	202	0.60	0.13-1.80	**<0.0001**	A&C; A&U

Kruskal-Wallis test was performed to compare Ashanti and Central and Upper West region, followed by Bonferroni post hoc test for pairwise analysis. P<0.05 was considered statistically significant (p-values of significant variable in bold print). TWBC: Total White Blood Cells; RBC: Red Blood Cells; HCT: Haematocrit; MCV: Mean Cell Volume, MCH: Mean Cell Haemoglobin; MCHC: Mean Cell Haemoglobin Concentration; RDW-CV: Red cell Distribution Width-Coefficient of Variation; Abs: Absolute.

## Data Availability

All relevant data are within the article. The original data used to support the findings of this study are available from the corresponding author upon reasonable request.
